# Impact of *Clostridium botulinum* genomic diversity on food safety

**DOI:** 10.1016/j.cofs.2016.09.006

**Published:** 2016-08

**Authors:** Michael W Peck, Arnoud HM van Vliet

**Affiliations:** 1 Gut Health and Food Safety, Institute of Food Research, Norwich Research Park, Norwich NR4 7UA, UK; 2 School of Veterinary Medicine, Faculty of Health and Medical Sciences, University of Surrey, Guildford GU2 7AL, UK

## Abstract

The deadly botulinum neurotoxin formed by *Clostridium botulinum* is the causative agent of foodborne botulism. The increasing availability of *C. botulinum* genome sequences is starting to allow the genomic diversity of *C. botulinum* Groups I and II and their neurotoxins to be characterised. This information will impact on microbiological food safety through improved surveillance and tracing/tracking during outbreaks, and a better characterisation of *C. botulinum* Groups I and II, including the risk presented, and new insights into their biology, food chain transmission, and evolution.

## Highlights

• *C. botulinum* Groups I and II form botulinum neurotoxin and cause foodborne botulism.
• Increased knowledge of *C. botulinum* Group I and II genomes and neurotoxin diversity.
• Impact on food safety via improved surveillance and tracing/tracking during outbreaks.
• New insights into *C. botulinum* biology, food chain transmission, evolution.

**Current Opinion in Food Science** 2016, **10**:52–59

This review comes from a themed issue on **Foodomics technologies**

Edited by **Stanley Brul**

For a complete overview see the Issue and the Editorial

Available online 30th September 2016

**http://dx.doi.org/10.1016/j.cofs.2016.09.006**

2214-7993/© 2016 The Authors. Published by Elsevier Ltd. This is an open access article under the CC BY license (http://creativecommons.org/licenses/by/4.0/).

## Introduction to *Clostridium botulinum* and foodborne botulism

Various molecular (including whole genome sequencing) and physiological approaches have shown that *Clostridium botulinum* is a diverse species that comprises four distinct groups of bacteria (*C. botulinum* Groups I–IV), that form the deadly botulinum neurotoxin. *C. botulinum* Groups I and II are associated with foodborne botulism and other forms of human botulism, *C. botulinum* Group III with botulism in animals, while *C. botulinum* Group IV has not been strongly associated with botulism. Some strains of *C. baratii* and *C. butyricum* also form botulinum neurotoxin and are associated with human botulism [1, 2, 3, 4, 5, 6•, 7•].

The botulinum neurotoxins are the most potent poison known, and foodborne botulism may be caused by consuming as little as 50 ng of neurotoxin [2, 8]. The neurotoxins are ∼150 kDa proteins that ultimately reach the nerve cell cytoplasm where they selectively cleave proteins involved in neurotransmitter (acetylcholine) release, bringing about a flaccid muscle paralysis [9, 10, 11, 12]. There are seven confirmed botulinum neurotoxins (types A–G), and many subtypes. Foodborne botulism is most commonly associated with neurotoxin types A, B or E, and occasionally with type F [1, 2, 3].

Foodborne botulism is a severe intoxication caused by consuming food containing botulinum neurotoxin formed by a strain of *C. botulinum* Group I or Group II, or more rarely by a strain of neurotoxigenic *C. baratii* or *C. butyricum*. Spores formed by these bacteria are ubiquitous in the environment, and to prevent foodborne botulism, it is necessary to identify and apply control measures that destroy spores, or prevent spore germination, cell multiplication and neurotoxin formation. A failure to apply suitable control measures has led to outbreaks of foodborne botulism with commercial and home-prepared foods. The commercial implications of foodborne botulism outbreaks can be significant [1, 2]. Strains of *C. botulinum* Group I (proteolytic *C. botulinum*) are highly proteolytic, mesophilic (minimum growth temperature of 12 °C), and form very heat resistant spores that are the target of the Botulinum cook (121 °C/3 min) given to low acid canned foods [1, 2]. A failure to apply the botulinum cook to canned or bottled foods has led to foodborne botulism, such as the large outbreak in 2015 associated with potato salad prepared using improperly home-canned potatoes [13]. *C. botulinum* Group II (non-proteolytic *C. botulinum*) is a psychrotrophic bacterium (minimum growth temperature of 3 °C), that ferments a range of carbohydrates, and forms spores of moderate heat resistance [1, 2]. Foodborne botulism outbreaks have been associated with temperature abuse of products intended to be stored chilled, such as vacuum packed fish, and the continued safe production of minimally processed chilled foods is a concern [14, 15, 16]. Details of further recent outbreaks of foodborne botulism involving *C. botulinum* Groups I and II are given in supplementary Table 1.

*C. botulinum* Group I strains possess up to three neurotoxin genes, and form up to three neurotoxins of type A, B and/or F. Strains with two neurotoxin genes form either one active toxin (e.g. type A(B) strains possess a type A and type B neurotoxin gene, but only form type A neurotoxin) or two active toxins (e.g. type Af strains form a greater amount of type A neurotoxin than type F neurotoxin). The neurotoxin gene(s) can be located on the chromosome or a large plasmid [17, 18, 19, 20, 21, 22]. *C. botulinum* Group II strains have a single neurotoxin gene, and form a single neurotoxin of types B, E or F (although strains that form type F toxin possess type B and E neurotoxin gene fragments [23]). The type B neurotoxin gene is located on a small plasmid, type F neurotoxin gene on the chromosome, while the type E neurotoxin gene is commonly present on the chromosome but can be located on a plasmid [23, 24, 25, 26•].

## Diversity of botulinum neurotoxins

Botulinum neurotoxins were initially separated into serotypes using specific antisera to neutralise neurotoxin in animal tests. However, sequencing of neurotoxin genes and derivation of the associated protein sequence has revealed important details on inter-serotype and intra-serotype variation. Smith *et al.* [27] analysed the sequence of 49 neurotoxins, and described two types of intra-serotype variation, firstly neurotoxin sequences that were identical or virtually identical, and secondly neurotoxin sequences that diverged by at least 2.6% in amino acid sequence and were designated as different subtypes. While this is a relatively arbitrary cut-off, it has provided the basis for a majority of subtype designations (e.g. [2, 4, 5, 27, 28, 29]), and has been proposed in a standardised approach to naming botulinum neurotoxin subtypes (MW Peck *et al.*, unpublished data).

**Figure 1:**
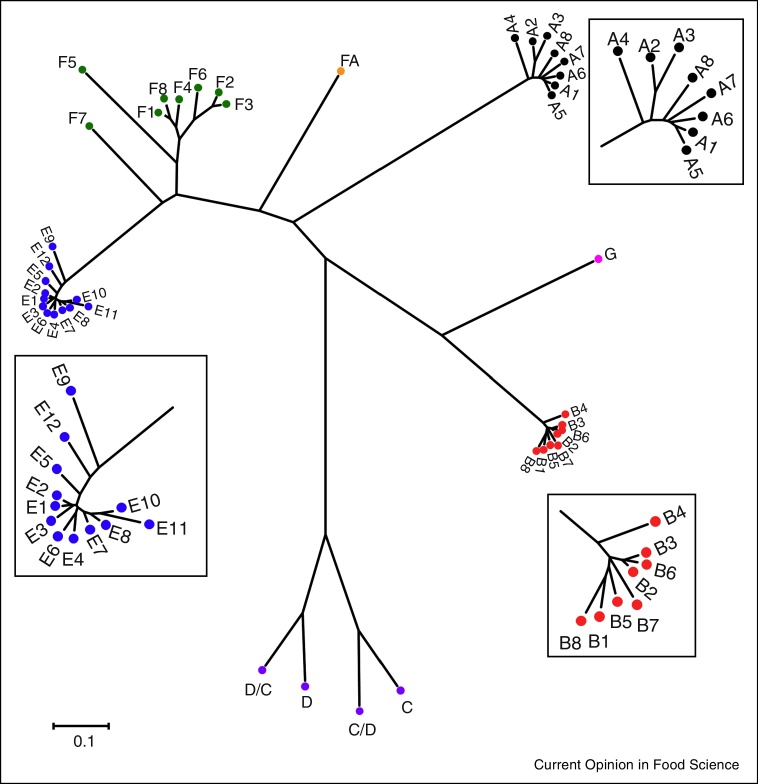
Phylogeny of botulinum neurotoxin subtypes. The sources of the sequences are given in supplementary Table 2. The toxin protein sequences were aligned with the Muscle algorithm, and the phylogenetic tree was generated using the Neighbour-Joining method, with the evolutionary distances computed using the JTT matrix-based method and the scale bar shows the number of amino acid substitutions per site. The tree is drawn to scale, with branch lengths in the same units as those of the evolutionary distances used to infer the phylogenetic tree. The analysis involved 41 amino acid sequences. All ambiguous positions were removed for each sequence pair. There were a total of 1409 positions in the final dataset. Alignment and phylogenetic analyses were conducted in MEGA7 [75]. The insets magnify the A, B and E subtypes for visualisation purposes, and do not match the scale bar.

A recent analysis of over 500 neurotoxin sequences (MW Peck *et al.*, unpublished data) identified 41 botulinum neurotoxin subtypes. A representative example of each is included in Figure 1. Each subtype displays a distinct pattern of inter-subtype and intra-subtype differences. Eight subtypes of type A neurotoxin are recognised (Figure 1) that show moderate inter-subtype differences (MW Peck *et al.*, unpublished data). There are also small intra-subtype differences (e.g. three intra-subtype A1 variants [6^•^]). Type B toxins and type E toxins are both less variable (Figure 1), with several type B subtypes and type E subtypes each differing by less than 2.6% in amino acid sequence, e.g. inter-subtype differences for subtypes B2, B3 and B6 are only 1.6–1.9%. Type F is a particularly variable neurotoxin, with significant inter-subtype differences, and subtypes F5 and F7 particularly dissimilar (Figure 1). Hybrids of type C and type D neurotoxin (type C/D or type D/C) are formed more frequently than type C or type D neurotoxin [30]. A highly novel neurotoxin (type FA (Figure 1), also known as type H and type HA) that contains regions of similarity to type A neurotoxin and type F neurotoxin has been recently identified and is being characterised [31, 32, 33, 34, 35, 36]. Hybrid neurotoxin subtypes have been identified, e.g. subtype A2 is a hybrid of subtypes A1 and A3 [37], subtype F6 is a hybrid of subtypes F1 and F2 [38]. Interestingly, subtype F6 neurotoxin is uniquely formed by Group II strains, while subtypes F1 and F2 are only formed by Group I strains [23, 39•]. This seems to be a rare example of neurotoxin genes crossing between Groups I and II, as no neurotoxin subtypes are known to be formed by both Group I and Group II strains (e.g. subtype B4 is exclusively formed by Group II strains, and the other seven type B subtypes are only formed by Group I strains [6•, 25]). Different neurotoxin types/subtypes display distinctive *in vitro* and *in vivo* toxicological properties (e.g. [40, 41, 42]).

The neurotoxin is associated with accessory proteins in various neurotoxin complexes. Genes encoding the neurotoxin and accessory proteins (e.g. non-toxic-non-haemagglutinin (NTNH)) are co-located in one of two conserved neurotoxin complex clusters (*ha* cluster or *orf-X* cluster) at one of several specific insertion sites on the chromosome or a plasmid [7•, 26•, 37, 38, 39•]. All type B and some type A neurotoxin genes are located in the *ha* cluster, that comprises genes encoding the neurotoxin, NTNH, three haemagglutinins, and a positive regulator (*botR*). Type A neurotoxin genes can also be located in the *orf-X* cluster, while type E and F genes are always present in the *orf-X* cluster. This cluster includes genes encoding the neurotoxin, NTNH, and four open reading frames of unknown function (*p47*, *orf-X1*, *orf-X2*, *orf-X3*). The gene (*botR*) encoding the positive regulatory protein is present in the *orf-X* gene cluster of Group I, but not Group II, strains [6•, 37, 39•].

The diversity of neurotoxin and accessory protein gene clusters is associated with the horizontal movement of genetic material via mobile elements (e.g. plasmid, phage), and various recombination/insertion events (e.g. simple recombination, insertions facilitated by transposases/insertion sequence elements, insertion of genetic material following targeting of homologous genes [21, 37, 38, 39•]). For example, it is postulated that a single recombination event has given rise to strains that possess an *ha* gene cluster and form subtype A1 neurotoxin, a very successful lineage that is frequently associated with foodborne botulism [21, 39•]. It is proposed that the *orf-X* cluster may be the ancestral subtype A1 neurotoxin gene cluster, and that the NTNH gene within this cluster has recombined with a NTNH gene within a Group I serotype B strain (within an *ha* cluster) to give a hybrid NTNH gene, and a downstream subtype A1 neurotoxin gene now in an *ha* gene cluster [21, 39•].

## Genomic diversity of *Clostridium botulinum* Group I

**Figure 2:**
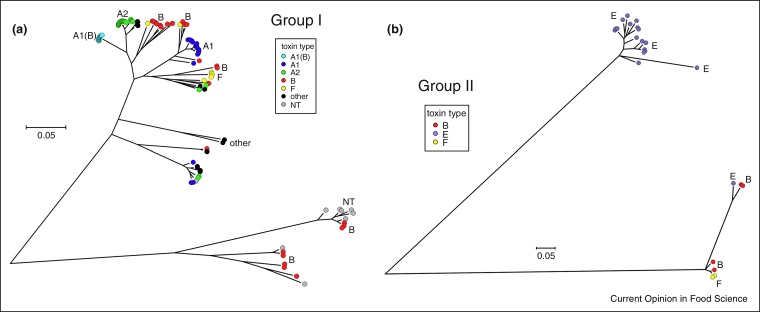
Distribution of botulinum neurotoxin types/subtypes in phylogenetic lineages of 108 *Clostridium botulinum* Group I isolates (panel a) and 24 *Clostridium botulinum* Group II isolates (panel b). Trees shown are based on comparison of core single nucleotide polymorphisms identified using parSNP [76], and plotted in the radial format using MEGA7 [75]. The *C. botulinum* Group I and Group II strains included in this Figure are listed in supplementary Table 3. Strains forming toxin types/subtypes A1, A2, A1(B), B, E and F are indicated in dark blue, green, light blue, red, purple and yellow, respectively. Strains forming toxin types/subtypes A3, Ab, Af, A5(B2′), Ba, Bf, and Bf/a are labelled as ‘others’ in black. Strains identified as ‘NT’ do not contain a botulinum neurotoxin gene.

Various approaches have been used to establish the diversity, and the evolutionary and phylogenetic relationship between strains of *C. botulinum* Group I. These have included comparative genomic indexing (using a DNA microarray), multi-locus sequence typing (MLST), pulsed-field gel electrophoresis (PFGE), multi-locus variable number tandem repeat analysis (MVLA), amplified fragment length polymorphism (AFLP), and comparisons of core genome single nucleotide polymorphisms (SNPs) following whole genome sequencing [2, 5, 6•, 7•, 22, 43•, 44, 45, 46, 47]. These approaches typically separate strains in up to about ten lineages or clusters (depending on strains tested and criterion applied). Figure 2 (panel a) shows the phylogenetic lineages of 108 Group I strains based on a comparison of core genome SNPs, with the neurotoxin types/subtypes indicated. Most clusters are dominated by strains that form the same neurotoxin(s); however, there is also evidence of neurotoxin genes (types/subtypes) in different genomic backgrounds, and of frequent horizontal movement of neurotoxin genes between clusters (Figure 2, panel a). Thus, for example, strains forming subtype A1 neurotoxin are primarily located in three clusters that represent; (i) strains with A1 toxin gene in *ha* cluster, (ii) strains with A1 toxin gene in *orf-x* cluster, and (iii) A1(B) strains. There are examples where strains forming subtype A1 neurotoxin are more closely related to strains forming other toxin types/subtypes than to other strains forming subtype A1 toxin (Figure 2, panel a). Strains forming type B neurotoxin have a diverse genomic background and are present in multiple clusters.

*C. sporogenes* is often used as a surrogate for *C. botulinum* Group I in food sterilisation tests [48]. However, it is now apparent that *C. sporogenes* is not simply a non-toxigenic version of *C. botulinum* Group I [5, 7•, 49•, 50]. Core genome SNP analysis reveals two clusters, that each contain strains of non-toxigenic *C. sporogenes* and Group I type B, distantly located from other Group I strains (lower two clusters in Figure 2, panel a). The neurotoxin gene is either subtype B2 (also present in strain 2345 [5]) or subtype B6; these subtypes are closely related (Figure 1) and are located on a plasmid [5, 7•, 39•, 49•, AT Carter *et al*., unpublished data]. The neurotoxigenic strains may represent examples of *C. sporogenes* strains that have acquired a type B neurotoxin gene, or possibly reflect neurotoxin gene instability as neurotoxin formation by strain 2345 is very unstable [AT Carter, unpublished data]. Elsewhere in this phylogenetic tree are Group I strains known to have lost their neurotoxin gene (Figure 2, panel a), and others are reported in the literature [7•, 49•, 51, 52]. Thus, Group I can be separated into strains that are ‘true *C. botulinum* Group I’, most of which (but not all) form neurotoxin, and strains that are ‘true *C. sporogenes*’, some of which form type B neurotoxin. This raises the question as to whether *C. sporogenes* is sufficiently different so as to be considered as a separate species from Group I; if this is the case it would represent a seventh distinct bacterium that is capable of forming botulinum neurotoxin. Weigand *et al*. [49^•^] reported that the average nucleotide identity for orthologous genes between the genomes of Group I and *C. sporogenes* was 93.4%, below the 95% cut-off frequently used for species differentiation [7•, 53], supporting the case for a separate species.

## Genomic diversity of *Clostridium botulinum* Group II

The same approaches described for *C. botulinum* Group I have been used to determine the genomic diversity of *C. botulinum* Group II. Group II is more diverse than Group I [2, 6•], and strains separate into two or three major clusters, depending on strains and criterion applied [4, 6•, 54•, 55]. A comparison of core genome SNPs of 24 Group II strains indeed reveals three lineages (Figure 2, panel b). The majority of type E strains are located in one cluster, which can be sub-divided further [54•, 55, 56]. It is likely that the type E strains arose from the insertion of the neurotoxin gene into genetically conserved bacteria, and that a series of recombination events then led to the type E neurotoxin subtypes [56].

The other two lineages are distantly separated from the type E cluster, but closely related to each other, and dominated by subtype B4 strains (Figure 2, panel b). One cluster includes strains that form subtype B4 or F6 neurotoxin. The F6 strains are highly clonal and seem to have arisen once following a series of events that has included the insertion of a 34 kb cassette that contains the subtype F6 neurotoxin gene into an ancestral type B strain, and the inactivation of the type B gene [23]. The other cluster includes subtype B4 strains and type E strains (Figure 2, panel b). This includes type E strains, isolated in France and Argentina [43•, 57, 58], that form neurotoxin subtype E9 or E12 (two closely related neurotoxins that are separate from other type E neurotoxins — Figure 1). Three further type E strains isolated from a food sample (whale meat) collected during a foodborne botulism outbreak are also closely related to type B strains [43•, 54•]. These unusual type E strains have presumably acquired neurotoxin genes by horizontal gene transfer, and represent rare examples of gene transfer between the type E cluster and the clusters dominated by subtype B4 strains (Figure 2, panel b). Interestingly, it is estimated that only ca. 0.8–1.2% of genes in Group II have been acquired by horizontal gene transfer, compared to ca. 4.5–6.8% of genes in Group I [3]. Williamson *et al*. [7^•^] also reported recombination to be less prevalent in Group II strains as compared to Group I strains. The genetic distance between the three Group II clusters (Figure 2, panel b) may hamper homologous recombination and hence reduce exchange of neurotoxin gene clusters and other genes.

The low frequency of horizontal neurotoxin gene transfer in Group II may explain why strains are not yet identified that form two neurotoxins (despite type B and type E neurotoxin genes being found on plasmids, and a mobile 24 kb cassette identified that contains the type E neurotoxin gene cluster [25, 26•]). Strains of Group II also often lose their neurotoxin genes [25, 54•, 55].

## Impact of genomic diversity on food safety

The cost of genome sequencing has fallen substantially since the first *C. botulinum* genome sequence was published less than ten years ago [17, 59]. The present availability of almost two hundred *C. botulinum* genome sequences has revealed much about the diversity *C. botulinum* Groups I and II. Each Group shows significant diversity, and may comprise multiple species, subspecies or genomovars [7^•^]. Additionally, it is likely that there is currently not a complete picture of this diversity.

There are several ways in which the information presently available can contribute to food safety. For example, PCR tests are being increasingly used in surveillance studies and investigation of foodborne botulism outbreaks [60, 61]. Our greater understanding of neurotoxin gene variability gives an increased value to these tests.

Botulism outbreaks are generally small, and whole genome sequencing and other molecular techniques have been used to compare outbreak associated strains. For example, strains isolated from the stool of individuals with botulism and a corresponding isolate from an implicated food were identical for several USA/Canadian foodborne botulism outbreaks [5, 29]. A molecular epidemiology study of infant botulism in California and elsewhere separated more than 1000 strains into 154 clades. Isolates from the patient and their home environment were frequently present in the same clade, indicating the probable source of infective spores [62]. A study of four time-coincident infant botulism cases in Australia revealed that the four Group I strains involved were related but not identical, suggesting that they had not come from a single source, but had diverged from a common ancestor many hundreds of years ago [63]. It is likely that whole genome sequencing and other molecular techniques will be of great value in future microbial forensic studies [64, 65].

Studies that have examined a diverse range of strains have generally reported there to be no relationship between phylogenetic position and association with botulism, geographic location, or isolation year (e.g. [4, 5, 7•, 22, 43•, 44, 55, 56, 66]). For example, lineages with Group I strains forming subtype A1 or A2 neurotoxins have been found throughout the world. However, it is becoming increasingly apparent that some lineages are associated with specific geographic locations. For example, lineages with type Af strains are dominant in Argentina [67], a geographical localisation of Group I clades has been identified in California [62], two dominant Group I type B clades have been identified in the Nordic environment [68], and it was possible to distinguish the neurotoxin-encoding plasmid in Group II subtype B4 strains isolated from European terrestrial environments and from marine (notably North America) environments [25]. Group II type E strains are frequently present in arctic and subarctic regions, but interestingly have now also been found in the southern hemisphere [57]. In a detailed study of Group II type E in northern Canada, strains forming subtype E3 neurotoxin were widespread in northern Canada with the exception of eastern Hudson Bay, subtype E10 strains were present along the shores of Ungava Bay and eastern Hudson Bay, and subtype E11 strains were restricted to the Koksoak River region of Nunavik [69^•^].

Bacterial genomics can make an important contribution to improved food safety and risk assessments [59, 64, 70, 71], including the foodborne botulism hazard. While the risk presented by Group I and Group II will always need to be considered separately, the question arises as to whether strains in certain lineages (and not necessarily certain neurotoxin types/subtypes) present a greater risk than others. It is known that strains in some lineages are more frequently associated with foodborne botulism than strains in other lineages. But are they more virulent (i.e. form more neurotoxin), do they survive and/or proliferate better in food environments, or is it simply greater opportunity? While it is not practical to consider individual strains, the use of molecular methods to associate clusters of strains that are closely related genetically (e.g. Figure 2) provides a possible way forward. For example, should the three *C. botulinum* Group II lineages (Figure 2, panel b) be considered together or separately? There is a need to improve risk assessments, but more complex assessments also need to be justified. Information is now becoming available to begin to inform these decisions. For example, a thousand-fold difference has been reported in the quantity of neurotoxin formed by type E strains [69^•^], and differences have been identified in carbohydrate utilisation patterns between the three Group II lineages, but not in growth response at chill temperature or at high NaCl concentrations [55]. Additionally, Wachnicka *et al*. [72] has reported on a relationship between lineage (and toxin type) and spore heat resistance. A systems level approach, both data mining and systems biology modelling, can provide a framework to understand complexity and hence to discover genetic determinants of features such as minimum growth temperature, neurotoxin formation and spore heat resistance [73, 74]. When challenge tests are carried out, it is customary to use a mixture of strains to reflect intra-species variability. Thus, for Group II, it might be appropriate to ensure that strains from all three lineages are included. Similar issues also apply to Group I, and in particular the selection of suitable non-toxigenic strains for thermal sterilisation tests.

A major future requirement is to increase understanding of the genomic diversity of *C. botulinum* Group I and Group II, the survival/proliferation of these bacteria in food, and the relationship between the two. This will provide new information on pathogen biology and transmission, and inform studies on pathogen evolution. Transcriptomic, proteomic and systems biology approaches will also use genomic data, and the findings will be equally important in future risk assessments, to extend understanding about mechanisms and control of phenotype, and may lead to the identification of novel intervention strategies.

## References and recommended reading

Papers of particular interest, published within the period of review, have been highlighted as:

• of special interest
•• of outstanding interest
