# Supplementary material for: Impact of Clostridium botulinum genomic diversity on food safety
Source: Curr Opin Food Sci. 2016 Aug;10:52–9. doi: 10.1016/j.cofs.2016.09.006 (PMC5181784; doi:10.1016/j.cofs.2016.09.006)
Supplement: Supplementary file 1 [file mmc1.docx]

**Supplementary Table 1 - Examples of recent outbreaks of foodborne botulism associated with *C. botulinum* Groups I and II**

| Toxin type | | Suspected or confirmed food | Number of cases (deaths) | Location, date | Reference |
| --- | --- | --- | --- | --- | --- |
| ***C. botulinum* Group I** | | | | |  |
| B | Fermented soybean | | 5 (0) | Thailand, 2010 | Wangroongsarb *et al.* (2014). Molecular characterisation of *Clostridium botulinum* isolates from foodborne outbreaks in Thailand, 2010. PLoS ONE 9, e77792. |
| A | Bean salad with home-canned beans | | 5 (1) | France, 2010 | Mazuet *et al*. (2012). Toxin detection in patients’ sera by mass spectrometry during two outbreaks of type A botulism in France. Journal of Clinical Microbiology 50, 4091-4094. |
| B | Commercial products; artichoke preserve, cream of vegetable soup (unlinked cases) | | 2 (0) | Italy, 2010 | Daminelli *et al*. (2011). Two unlinked cases of foodborne botulism in Italy at the beginning of 2010. New Microbiol. 34, 287-290. |
| A | Commercial curry sauce in jar | | 3 (0) | UK, 2011 | Browning *et al*. (2011). An outbreak of food-borne botulism in Scotland, United Kingdom, November 2011. Euro Surveill 16, pii=20036. |
| A | Commercial (artisan) ground green olive paste | | 9 (0) | France, 2011 | Pingeon *et al*. (2011). Two outbreaks of botulism associated with consumption of green olive paste, France, September 2011. Euro Surveill 16, pii=20035. |
| A | Home-canned homogenised turkey | | 1 (1) | Italy, 2011 | Lonati *et al*. (2011). Fatal course of foodborne botulism in eight-month old infant. Pediatric Reports 3, e31. |
| B | Commercial olives stuffed with almonds (from Italy) | | 2 (1) | Finland, 2011 | Jalava *et al*. (2011). Two cases of food-borne botulism in Finland caused by conserved olives, October 2011. Euro Surveill 16, pii=20034. |
| A | Illicit prison alcohol “pruno”, made with potatoes | | 8 (0) | USA, 2011 | Thurston *et al.* (2012). Botulism From Drinking Prison-Made Illicit Alcohol — Utah 2011. Morbidity and Mortality Weekly Reports 61, 782-784. |
| A | Commercial potato soup (2 unlinked cases) | | 2 (0) | USA, 2011 | Seaman *et al.* (2011). Botulism Caused by Consumption of Commercially Produced Potato Soups Stored Improperly --- Ohio and Georgia. Morbidity and Mortality Weekly Reports 60, 890-891. |
| A | Home-made olive and tuna pate (?) | | 2 (0) | Spain, 2011 | Lafuente *et al*. (2013). Two simultaneous botulism outbreaks in Barcelona: *Clostridium baratii* and *Clostridium botulinum*. Epidemiol. Infect. 141, 1993-1995. |
| B | Home-made fermented crabmeat with salt (?) | | 2 (0) | Thailand, 2012 | Wangroongsarb *et al.* (2013). An outbreak of foodborne botulism in Surat Thani Province Thailand, 2012. Jpn. J. Infect. Dis. 66, 353-354. |
| A | Commercial vacuum packed sweet adzuki bean soup | | 2 (0) | Japan, 2012 | Momose *et al.* (2014). Food-borne botulism in Japan in March 2012. International Journal of Infectious Diseases 24, 20-22. |
| A | Illicit prison alcohol “pruno”, made with potatoes (2 outbreaks) | | 12 (0) | USA, 2012 | Briggs *et al*. (2013). Botulism From Drinking Prison-Made Illicit Alcohol — Arizona, 2012. Morbidity and Mortality Weekly Reports 62, 88-90. |
| A | Home-canned beets | | 3 (1) | USA, 2012 | [www.cdc.gov/nationalsurveillance/botulism-surveillance.html](http://www.cdc.gov/nationalsurveillance/botulism-surveillance.html) (accessed June 2016). |
| B | Home-preserved turnip tops in oil | | 1 (0) | Italy, 2013 | Anniballi *et al.* (2015). Foodborne botulism associated with home-preserved turnip tops in Italy. Ann. Ist. Super. Sanita 51, 60-61. |
| A | Commercial smoked ribs | | 12 (0) | China, 2013 | Feng *et al.* (2015). Two-family outbreak of botulism associated with consumption of smoked ribs in Sichuan province, China. International Journal of Infectious Diseases 30, 74-77. |
| B | Commercial jarred pesto | | 2 (0) | USA, 2014 | Burke *et al.* (2016). Outbreak of foodborne botulism associated with improperly jarred pesto – Ohio and California, 2014. Morbidity and Mortality Weekly Reports 65, 175-177. |
| A | Potato salad with home-canned potatoes | | 29 (1) | USA, 2015 | McCarty *et al*. (2015). Large outbreak of botulism associated with a church potluck meal – Ohio, 2015. Morbidity and Mortality Weekly Reports 64, 802-803. |
| ***C. botulinum* Group II** | | | | |  |
| B | | Home-prepared ham (2 outbreaks) | 10 (0) | France, 2010 | Mazuet *et al*. (2014). Le botulisme humain en France, 2010-2012. Bull Epidémiol Hebd 106-114. |
| E | | Commercial vacuum packed bean curd | 1 (0) | Taiwan, 2011 | Lai *et al.* (2011). Foodborne botulism type E intoxication associated with dried bean curd: first case report in Taiwan. Acta Neurologica Taiwanica 20, 138-141. |
| E | | Salmon eggs | 3 (0) | USA, 2011 | [www.cdc.gov/nationalsurveillance/botulism-surveillance.html](http://www.cdc.gov/nationalsurveillance/botulism-surveillance.html) (accessed June 2016). |
| E | | Commercial salted fish (Faseikh) | 3 (0) | Canada, 2012 | Walton *et al.* (2014). Outbreak of type E foodborne botulism linked to traditionally prepared salted fish in Ontario, Canada. Foodborne Pathogens and Disease 11, 830-834. |
| B | | Home-prepared ham | 2 (0) | France, 2012 | Mazuet *et al*. (2014). Le botulisme humain en France, 2010-2012. Bull Epidémiol Hebd 106-114. |
| E | | Partially fermented Eider duck | 5 (2) | Greenland, 2013 | Hammer *et al*. (2015). Fatal outbreak of botulism in Greenland. Infectious Diseases 47, 190-194. |
| E | | Aged seal meat/blubber | 1 (1) | Greenland, 2013 | Leth (2014). Botulism in Greenland. Forensic Science international 238, e1-e2. |
| E | | Vacuum packed whitefish | 1 (0) | Sweden, 2014 | Personne & Skagius (2015). Botulism caused by vacuum packed whitefish (*Corregonus lavaretus*). Clinical Toxicology 53, 338-339. |
| E | | Stinkheads (2 outbreaks) | 4 (1) | USA, 2014 | [www.cdc.gov/nationalsurveillance/botulism-surveillance.html](http://www.cdc.gov/nationalsurveillance/botulism-surveillance.html) (accessed June 2016). |
| E | | Seal oil | 3 (0) | USA, 2014 | [www.cdc.gov/nationalsurveillance/botulism-surveillance.html](http://www.cdc.gov/nationalsurveillance/botulism-surveillance.html) (accessed June 2016). |
