# Supplementary material for: Impact of Clostridium botulinum genomic diversity on food safety
Source: Curr Opin Food Sci. 2016 Aug;10:52–9. doi: 10.1016/j.cofs.2016.09.006 (PMC5181784; doi:10.1016/j.cofs.2016.09.006)
Supplement: Supplementary file 2 [file mmc2.pptx]

## Slide 1
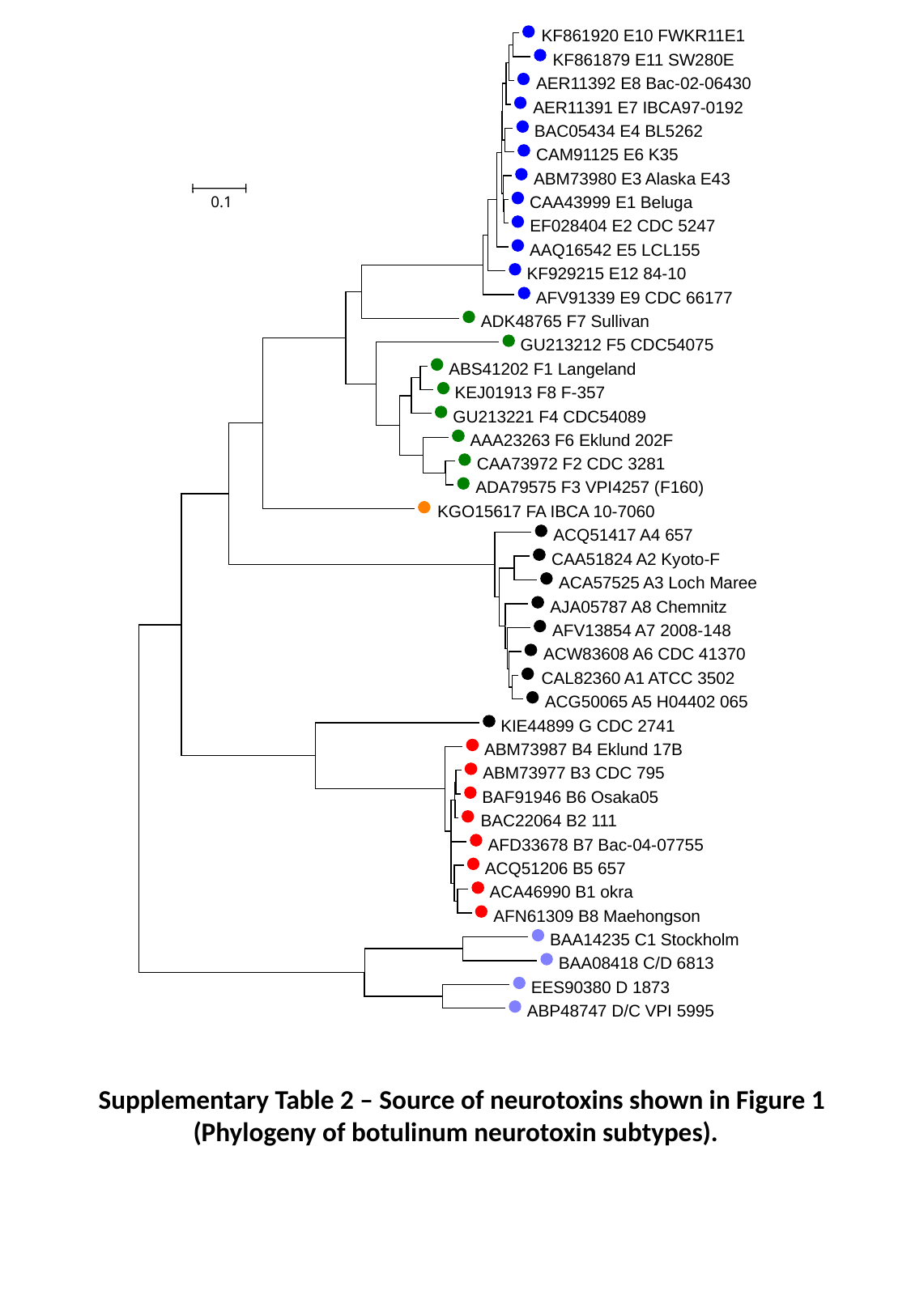

KF861920 E10 FWKR11E1
 KF861879 E11 SW280E
 AER11392 E8 Bac-02-06430
 AER11391 E7 IBCA97-0192
 BAC05434 E4 BL5262
 CAM91125 E6 K35
 ABM73980 E3 Alaska E43
0.1
 CAA43999 E1 Beluga
 EF028404 E2 CDC 5247
 AAQ16542 E5 LCL155
 KF929215 E12 84-10
 AFV91339 E9 CDC 66177
 ADK48765 F7 Sullivan
 GU213212 F5 CDC54075
 ABS41202 F1 Langeland
 KEJ01913 F8 F-357
 GU213221 F4 CDC54089
 AAA23263 F6 Eklund 202F
 CAA73972 F2 CDC 3281
 ADA79575 F3 VPI4257 (F160)
 KGO15617 FA IBCA 10-7060
 ACQ51417 A4 657
 CAA51824 A2 Kyoto-F
 ACA57525 A3 Loch Maree
 AJA05787 A8 Chemnitz
 AFV13854 A7 2008-148
 ACW83608 A6 CDC 41370
 CAL82360 A1 ATCC 3502
 ACG50065 A5 H04402 065
 KIE44899 G CDC 2741
 ABM73987 B4 Eklund 17B
 ABM73977 B3 CDC 795
 BAF91946 B6 Osaka05
 BAC22064 B2 111
 AFD33678 B7 Bac-04-07755
 ACQ51206 B5 657
 ACA46990 B1 okra
 AFN61309 B8 Maehongson
 BAA14235 C1 Stockholm
 BAA08418 C/D 6813
 EES90380 D 1873
 ABP48747 D/C VPI 5995
Supplementary Table 2 – Source of neurotoxins shown in Figure 1 (Phylogeny of botulinum neurotoxin subtypes).
